# Natural Selection Drives AT-Biased Codon Usage in Mitochondrial Genomes of Early-Diverging *Conidiobolus* Fungi (Zoopagomycota)

**DOI:** 10.3390/jof12040231

**Published:** 2026-03-24

**Authors:** Yanan Cao, Xianli Guo, Jialin Yang, Xiyue Yan, Yanping Xu, Qiang Li, Zehou Liu

**Affiliations:** 1 Key Laboratory of Coarse Cereal Processing, Ministry of Agriculture and Rural Affairs, School of Food and Biological Engineering, Chengdu University, Chengdu 610106, China; caoyanan@cdu.edu.cn (Y.C.); 18782136172@163.com (X.G.); 13882388102@163.com (J.Y.); 18613235090@163.com (X.Y.); xuyp0422@163.com (Y.X.); 2 Crop Research Institute, Sichuan Academy of Agricultural Sciences, Chengdu 610066, China

**Keywords:** codon usage bias (CUB), mitochondrial genome, natural selection, phylogenomic discordance, *Conidiobolus*

## Abstract

Codon usage bias (CUB) in mitochondrial genomes reflects evolutionary forces such as mutation, selection, and genetic drift, yet its dynamics in early-diverging fungal lineages like *Conidiobolus* (Zoopagomycota) remain unclear. This study systematically analyzed mitochondrial core protein-coding genes (PCGs) from eight *Conidiobolus* species to elucidate the drivers of CUB and phylogenomic patterns. Nucleotide composition revealed pronounced AT richness (73.32% ± 3.38%) and low GC3 (13.40% ± 5.11%), indicating a preference for A/T-ending codons. Neutrality and ENC-GC3s plots demonstrated that natural selection, rather than mutation pressure, predominantly shaped codon bias, supported by weak GC12-GC3 correlations (slopes: 0.037–0.335) and significant ENC deviations from mutation-driven expectations. PR2-bias analysis further highlighted a strong bias toward A over T and C over G. Correspondence analysis linked major codon usage variations to GC3s, CAI, and FOP indices. Phylogenetic reconstructions based on relative synonymous codon usage (RSCU) and concatenated mitochondrial sequences revealed discordant topologies, particularly in the placement of *C. polytocus* and *C. polyspermus*, suggesting divergent evolutionary trajectories. Optimal codon analysis identified species-specific preferences dominated by A/T termini. These findings underscore natural selection as the primary force driving AT-biased mitochondrial CUB in *Conidiobolus*, while phylogenomic discordance highlights complex evolutionary pressures in this ecologically diverse fungal genus. This study provides foundational insights into mitochondrial genome evolution and codon adaptation mechanisms in early-diverging fungi.

## 1. Introduction

The genetic information encoded in DNA undergoes transcription to produce a sequence of 20 amino acids by the central dogma [1]. Among the 64 codons in DNA, 61 code for standard amino acids, while the remaining three function as termination signals for translation [2]. Most amino acids are specified by multiple synonymous codons, except for tryptophan and methionine, each represented by a single codon [3]. The redundancy in codon usage, termed degeneracy, allows for variability in the frequency of synonymous codons used by different organisms and genes, a phenomenon known as codon usage bias (CUB) [4]. The alteration of a synonymous codon does not affect the amino acid sequence due to mutations occurring in the gene’s coding region, particularly in the second or third nucleotide of the codon [5]. These synonymous mutations, referred to as ‘silent mutations,’ do not impact protein function but contribute to variations in codon usage across organisms [6]. Codon bias is influenced by mutation patterns, with certain codons being more prone to mutations [7], thereby being preserved by selection pressure [8]. Factors such as GC heterogeneity [9] and GC-biased gene conversion also play a role in shaping codon bias through local recombination rates [10]. The evolution of synonymous codons is governed by mutations, natural selection, and genetic drift [11,12], ultimately impacting gene translation efficiency and genome evolution [13]. Codon bias is ascribed to nucleotide bias [14] and the rate of point mutations [15], elucidating differences in codon usage among different species [16]. The theory of natural selection posits that synonymous mutations affecting organismal adaptability are the driving force behind variations in codon usage during genome or gene evolution [17,18].

Codon bias impacts various cellular processes, including mRNA stability, transcription, translation efficiency, accuracy, protein expression, structure, function, and cotranslational folding [19,20]. It can affect transcription levels through its influence on chromatin structure and mRNA folding, as well as regulate translation efficiency by controlling the translation elongation rate [21,22,23]. Codon bias reflects genome adaptations to transcription and translation mechanisms and is essential for studying gene molecular evolution, enabling the selection of gene sequences without changing amino acids [24,25,26]. Similar codon usage among closely related organisms may suggest horizontal gene transfer and evolutionary relationships [27,28]. Genes encoding highly expressed proteins typically use optimal codons, highlighting the value of codon optimization in genetic engineering and recombinant DNA technology to enhance protein expression [29,30]. The comprehensive analysis of codon bias, facilitated by high-throughput sequencing technology, contributes to understanding species evolution, environmental adaptation, and genetics [31,32,33]. However, the genetic characteristics of codon bias in fungal species from Zoopagomycota remain unknown.

The mitochondrial genome plays a pivotal role in cellular energy production and evolutionary studies, showcasing distinct patterns of codon usage bias (CUB) influenced by mutational pressure, natural selection, and genetic drift [34,35]. Mitochondrial genomes offer valuable insights into evolutionary processes due to their elevated mutation rates, lack of recombination, and potential co-evolution with nuclear genomes [36,37,38]. Fungi, characterized by compact genomes and uniparental inheritance, present an intriguing subject for investigating mitochondrial CUB, particularly in Zoopagomycota fungi [39]. Among the Zoopagomycota, the *Conidiobolus* genus, a core taxon of the Ancylistaceae family, stands out. *Conidiobolus* species are widely distributed in various habitats, from soils to plant residues, across temperate to tropical regions [40,41,42,43]. For instance, *C. mycophagus* is an obligate nematode parasite, *C. polytocus* infects a broad range of hosts, *C. taihushanensis* has a GC-rich genome and is saprophytic, and *C. polyspermus* is a lignin-degrading fungus [44,45,46]. Moreover, *C. rhodosporus* and *C. chloridiosporus* exhibit strong adaptability to adverse conditions, while *C. lichenicola* is a unique lichen symbiont. The diverse morphology, life history, and ecological roles of *Conidiobolus* fungi encompass various survival strategies, including pathogen parasitism, saprophytic decomposition, symbiotic relationships, and stress adaptation, offering a comprehensive research model for unraveling the evolutionary mechanisms underlying codon usage preferences [44,45,46]. However, the exploration of mitochondrial CUB in *Conidiobolus* and even Zoopagomycota remains insufficient [39,47].

In this study, we systematically analyzed and compared the codon usage characteristics in the mitochondrial core protein-coding genes of eight *Conidiobolus* species: *C. heterosporus*, *C. taihushanensis*, *C. rhysosporus*, *C. polytocus*, *C. lichenicola*, *C. polyspermus*, *C. mycophagus*, and *C. chlamydosporus*. A phylogenetic tree of *Conidiobolus* species was constructed based on the relative synonymous codon usage of mitochondrial core genes and compared with a tree constructed using core protein-encoding gene sequences. This study presents the first comprehensive analysis of mitochondrial CUB in *Conidiobolus*, utilizing mitochondrial genome sequences from diverse species to elucidate the roles of mutational bias, natural selection, and phylogenetic inertia.

## 2. Materials and Methods

### 2.1. Sequence Processing

Eight complete mitochondrial genomes from the *Conidiobolus* genus are accessible in the National Center for Biotechnology Information (NCBI) database (https://www.ncbi.nlm.nih.gov/nuccore/, accessed on 1 April 2025). The core protein-coding genes were extracted using the following accession numbers: NC_040967, MZ436173, MZ286627, MZ436172, MZ436169, MZ436171, MZ436170, and MZ436168. To minimize stochastic effects in codon usage analysis, genes shorter than 100 codons (300 nucleotides) were excluded, resulting in 11 common core protein-coding genes for subsequent analysis in each *Conidiobolus* mitochondrial genome: *atp6*, *cob*, *cox1*, *cox2*, *cox3*, *nad1*, *nad2*, *nad3*, *nad4*, *nad5*, and *nad6* [48].

### 2.2. Codon Usage Indices

The GC3s parameter determines the proportion of codons containing guanine and cytosine at the third synonymous positions, excluding methionine, tryptophan, and termination codons [49]. The codon adaptation index (CAI) is a widely used metric for assessing bias towards codons favored in highly expressed genes, with a range of 0 to 1.0 indicating the frequency of synonymous codon usage. The codon bias index (CBI) is a standard for evaluating gene expression, reflecting the prevalence of optimal codons in highly expressed genes. The frequency of optimal codons (FOP) is determined by the ratio of optimal codons to total synonymous codons in a gene. The effective number of codons (ENC) indicates the diversity of codons used in a gene, ranging from 20 to 61. An ENC value of 20 implies the use of only one codon per amino acid, while 61 suggests equal usage of all codons. ENC values below 35 indicate strong codon usage bias, whereas values above 35 indicate a weak preference for codon usage. The Relative Synonymous Codon Usage (RSCU) was determined by analyzing the amino acids encoded by the same codons and their respective probabilities. RSCU values >1 indicate positive codon bias, <1 indicate negative bias, and =1 indicate random usage. The general average hydropathicity (GRAVY) values were calculated by summing the hydropathy values of all amino acids in polymerase gene sequences and multiplying by the number of residues, ranging from −2 to 2, where positive values represent hydrophobic and negative values represent hydrophilic proteins. The aromaticity (AROMO) value reflects the frequency of aromatic amino acids (Phe, Tyr, Trp). These values were all computed using CodonW 1.4.2 [50].

### 2.3. Neutrality Plot Analysis

The neutrality plot (GC12 vs. GC3) elucidates the equilibrium between mutation and selection in codon bias formation. GC12 represents the average GC content at the first and second codon positions (GC1 and GC2), while GC3 indicates the GC content at the third position. A strong statistical correlation between GC12 and GC3 implies that mutation pressure is the primary evolutionary force, whereas the absence of correlation suggests that natural selection is the predominant driving force in the species being studied.

### 2.4. ENC-GC3s Plot Analysis

The ENC-GC3s plot, commonly used to evaluate the influence of mutation versus other factors like natural selection on codon usage in a gene, displays ENC values on the *y*-axis and GC3s values on the *x*-axis. A specific formula was employed to derive the expected curve on this plot. Clustering of data points around this curve suggests mutation pressure as the main determinant of codon bias. Conversely, significant deviation from the expected curve indicates the involvement of additional factors, such as natural selection, in shaping codon bias.

$$ENC_{exp}=2+GC3_{s}+\frac{29}{GC3_{s}^{2}+{\left(1-GC3_{s}\right)}^{2}}$$

Further analysis was conducted on the discrepancies between the anticipated and observed values of ENC, indicated by the *ENC_Ratio_* index, which quantifies the degree of deviation between these values.

$$ENC_{Ratio}=\frac{ENC_{exp}-ENC_{obs}}{ENC_{exp}}$$

### 2.5. PR2-Bias Plot Analysis

We performed the Parity Rule 2 bias (PR2-Bias) plot analysis by comparing the ratio of A3/(A3 + U3) to G3/(G3 + C3). The central point of the plot signifies A=T and C=G, denoting the absence of usage bias in the codon, with the vectors emanating from this point illustrating the extent and direction of the gene bias.

### 2.6. Correspondence Analysis

Correspondence analysis (COA) is a prevalent multivariate statistical technique for studying codon usage patterns. By projecting all genes into a 59-dimensional hyperspace (excluding Met and Trp out of the total 61 codons), this method facilitates the examination of primary trends in codon usage variability within the core CDS of *Conidiobolus*. Codons are then positioned along the axes according to their RSCU values.

### 2.7. Determination of Optimal Codons

Genes were arranged based on their Effective Number of Codons (ENC) values, with 10% of genes selected from both extremes to establish high and low expression gene pools. High expression codons were distinguished by computing the Difference value (D-value) between the Relative Synonymous Codon Usage (RSCU) of the two pools (ΔRSCU), with codons having a ΔRSCU > 0.08 identified as high expression. Codons surpassing an RSCU of 1 were deemed high-frequency, while the optimal codon was characterized by meeting the criteria of ΔRSCU > 0.08 and RSCU > 1.

### 2.8. Phylogenetic Analysis

Phylogenetic relationships among *Conidiobolus* species were assessed through the comparison of codon usage and mitochondrial sequence-based methods. RSCU values of eight *Conidiobolus* species were analyzed using SPSS v19.0 software, employing hierarchical clustering to construct a relationship tree. Specifically, RSCU values for each species (an 11 genes × 59 synonymous codons matrix) were standardized by converting to Z-scores across genes to minimize scale effects. A Euclidean distance matrix was then calculated to quantify pairwise dissimilarity in codon usage patterns. Finally, a dendrogram was generated using the unweighted pair group method with arithmetic mean (UPGMA) clustering algorithm in SPSS v19.0. Phylogenetic trees were generated from combined mitochondrial gene datasets following established protocols [51,52]. Mitochondrial genes were aligned using MAFFT v7.037 [53], concatenated with Sequence Matrix v1.7.8 [54], and evaluated for conflicts through a partition homogeneity test. Partition Finder 2.1.1 determined the optimal model for partitioning and evolution [55]. Bayesian inference analysis was conducted using MrBayes v3.2.6 [56], with two independent 2 × 10^6^ generation chains and sampling every 100 generations. Stationarity was considered achieved when the sample size exceeded 100, and the scale reduction factor approached 1.0. The initial 25% of samples were discarded as burn-in, and the remaining trees were used to calculate Bayesian posterior probabilities in a 50% majority-rule consensus tree.

## 3. Results

### 3.1. Nucleotide Composition of Conidiobolus Core PCGs

Eleven mitochondrial core protein-coding genes (PCGs) were chosen from eight *Conidiobolus* species for codon usage analysis. The average length of these core PCGs ranged from 354 bp to 1966 bp, with *nad3* being the shortest and *nad5* being the longest. Among the 11 core PCGs, nine showed variations in sequence length across the *Conidiobolus* species, while *cox3* and *nad3* maintained consistent lengths across all species. The *nad2* gene displayed the most significant length difference, with a 294 bp gap between the longest and shortest sequences. Analysis of base composition in the 11 core PCGs revealed a predominance of A bases at 37.06%, followed by T bases at 36.26%. G and C bases were less abundant, averaging 14.70% and 11.98%, respectively. The GC content of the 11 core PCGs ranged from 20.09% to 32.27%, with *nad3* having the lowest GC content and *cox1* having the highest.

### 3.2. Codon Usage Analysis

**Figure 1:**
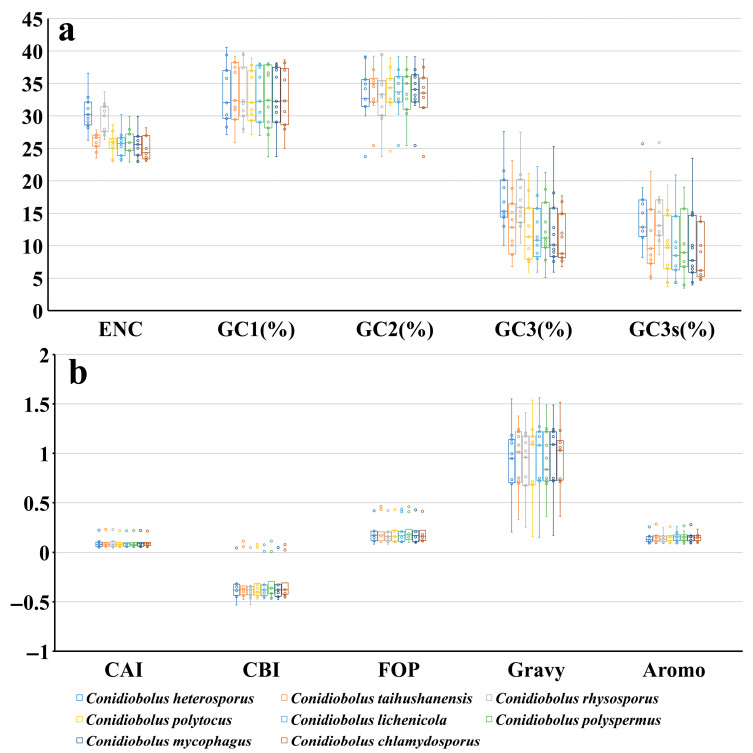
Codon usage indicators of 11 mitochondrial core protein coding genes in different *Conidiobolus* strains. (**a**) and (**b**) respectively express different indicators. Horizontal representation of median in box plot.

The GC contents of GC1, GC2, and GC3 in the 11 core protein-coding genes (PCGs) of 8 *Conidiobolus* species were 34.06%, 33.58%, and 13.40%, respectively (Figure 1a). The average GC3 value of the 11 PCGs in these species was 13.40%, indicating a preference for A or T bases at the end of mitochondrial core PCG codons in *Conidiobolus*. Analysis of the A3, T3, G3, and C3 indexes of the 11 core PCGs revealed a higher tendency for codons to end with A, followed by T, G, and C, with values of 57.78%, 28.82%, 6.76%, and 6.64%, respectively. Calculation of the Codon Adaptation Index (CAI) values for the 11 core PCGs in the 8 *Conidiobolus* species showed a range from 0.052 to 0.226 (Figure 1b). Notably, *nad4* exhibited the lowest CAI value, whereas *nad6* displayed the highest, indicating a pronounced codon bias in the *nad4* gene. CAI values were uniformly low across all species, ranging from 0.052 to 0.226 (Figure 1b). *C. taihushanensis* and *C. polyspermus* tended to have relatively higher CAI values, while *C. heterosporus* has low CAI value, indicating significant codon bias in its mitochondrial core PCGs. The Codon Bias Index (CBI) values of the 11 core PCGs range from −0.469 to 0.076, with *nad4* displaying the lowest and *nad6* the highest values. Within *Conidiobolus* species, CBI values range from −0.359 to −0.305, with *C. rhysosporus* having the lowest and *C. polyspermus* the highest values. The Frequency of Optimal Codons (FOP) values among the 11 core PCGs range from 0.101 to 0.438, with *nad4* having the lowest and *nad6* the highest values. *C. rhysosporus* exhibits the lowest FOP value among *Conidiobolus* species, while *C. polyspermus* has the highest. The Grand Average of Hydropathicity (GRAVY) values for the 11 core PCGs in *Conidiobolus* species indicate that all are hydrophobic. The Average Range of Molecular Orientation (AROMO) values range from 0.087 to 0.260, with *nad6* having the highest and *atp6* the lowest value. AROMO values exhibit minimal variation among different *Conidiobolus* species, with an average of 0.146.

### 3.3. Codon Usage Correlation Analysis

**Figure 2:**
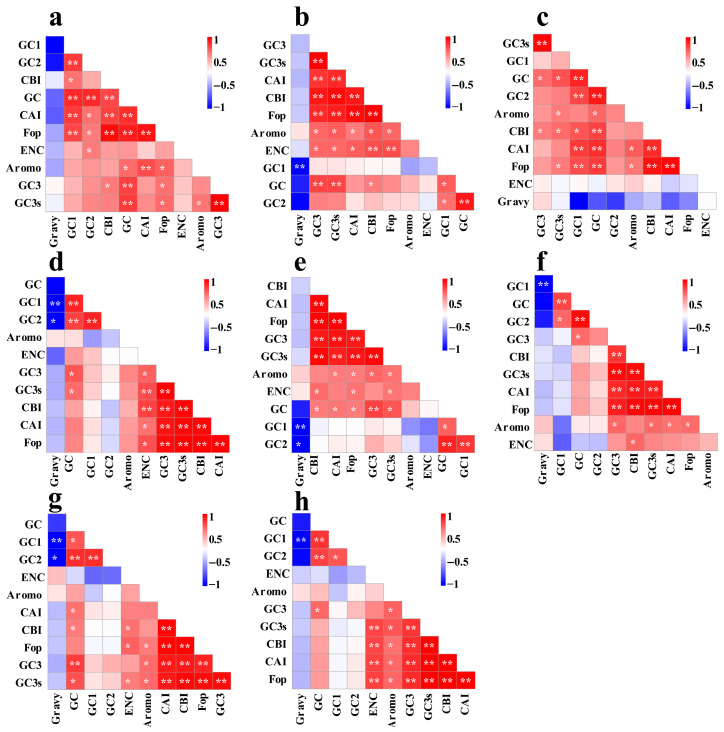
Pearson’s correlation analysis heatmap of different codon usage indicators of 8 *Conidiobolus* strains. The color of the color block changes from blue to red, indicating that the correlation index is increasing. One asterisk indicates a significant correlation between different indicators at the *p* < 0.05 level, while two asterisks indicate a significant correlation between different indicators at the *p* < 0.01 level. The 8 *Conidiobolus* species are *C. heterosporus*, *C. taihushanensis*, *C. rhysosporus*, *C. polytocus*, *C. lichenicola*, *C. polyspermus*, *C. mycophagus*, and *C. chlamydosporus* from (**a**–**h**).

The GC1 content in mitochondrial codons exhibited a significant correlation with GC2 and overall GC content across all eight *Conidiobolus* species, indicating its influence on codon usage in *Conidiobolus* mitochondrial protein-coding genes (PCGs) (Figure 2). Additionally, a significant negative correlation was observed between GC1 content and Gravy values in six out of eight *Conidiobolus* species. The GC2 content was significantly associated with the species’ overall GC content, suggesting its impact on the species’ GC composition. Furthermore, GC3 content showed significant associations with the Codon Bias Index (CBI), GC, and GC3s indices in the eight *Conidiobolus* species. The GC content of mitochondrial codons was significantly correlated with the Codon Adaptation Index (CAI), CBI, and Fop index in four out of the eight *Conidiobolus* species (*p* < 0.05). Moreover, significant correlations were found between the CAI index and the CBI and FOP indices in all eight *Conidiobolus* species (*p* < 0.05).

### 3.4. Neutrality Plot Results

**Figure 3:**
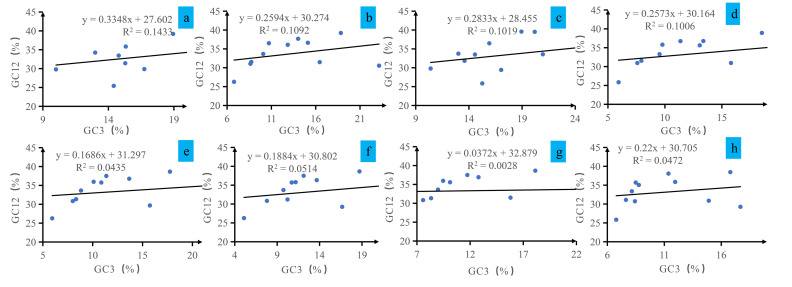
Neutrality plot analysis of GC12 and the third codon position (GC3) for the entire coding DNA sequence of 8 *Conidiobolus* strains. The 8 *Conidiobolus* species are *C. heterosporus*, *C. taihushanensis*, *C. rhysosporus*, *C. polytocus*, *C. lichenicola*, *C. polyspermus*, *C. mycophagus*, and *C. chlamydosporus* from (**a**–**h**).

The relationship between GC12 and GC3 in *Conidiobolus* mitochondrial codons was examined through neutrality plot analysis (Figure 3). The GC12 content varied from 25.42% to 39.90%, while the GC3 content ranged from 5.08% to 27.62%, demonstrating significant variability. Regression analysis indicated slopes between 0.037 and 0.335, suggesting a weak positive association between GC12 and GC3 content. The R2 values of the regression lines fell within the range of 0.0028 to 0.1433. The weak correlation between GC12 and GC3 indicates that variation in codon usage cannot be explained by GC3-driven mutational pressure alone. This deviation from neutral expectations is consistent with the influence of additional factors, such as natural selection on synonymous codons or functional constraints linked to amino acid composition in the highly AT-rich mitochondrial background.

### 3.5. ENC-GC3s Plot Results

**Figure 4:**
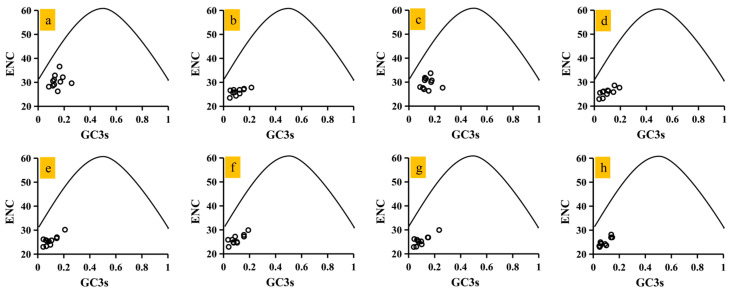
ENC-GC3 plot analysis of 11 core PCGs in 8 *Conidiobolus* strains. The solid line represents the expected curve when codon usage bias is affected only by mutation pressure. Circles represent the data points of 11 PCGs. The 8 *Conidiobolus* species are *C. heterosporus*, *C. taihushanensis*, *C. rhysosporus*, *C. polytocus*, *C. lichenicola*, *C. polyspermus*, *C. mycophagus*, and *C. chlamydosporus* from (**a**–**h**).

The mean effective number of codons (ENC) for the 11 core protein-coding genes (PCGs) detected fell within the range of 24.83 to 30.32, with an average ENC value of 26.80 (Figure 1a). All core PCGs exhibited ENC values below 35, indicating significant codon usage bias. Notably, the ENC values for the eight *Conidiobolus* species varied from 24.85 to 30.48, highlighting a distinct preference for codon usage in *Conidiobolus* mitochondrial genes. An ENC plot was constructed to evaluate the impact of GC3s on codon bias in *Conidiobolus* (Figure 4). The observation that all data points fall below the expected curve suggests that mutational pressure (as reflected by GC3s) is not the sole determinant of codon bias. The significant deviation (ENC~Ratio~: 13.13–43.92%) could be attributed to the combined effects of natural selection, amino acid conservation, and/or strand-specific mutation biases that are characteristic of AT-rich mitochondrial genomes. The ENC_Ratio_ was calculated for mitochondrial core genes in *Conidiobolus* to measure the difference between observed and expected ENC values. The average ENC_Ratio_ values for all core PCGs ranged from 13.13% to 43.92% (Figure S1), indicating lower observed values compared to the expected values, with GC3s values influencing the expected ENC values.

### 3.6. PR2-Bias Plot Results

**Figure 5:**
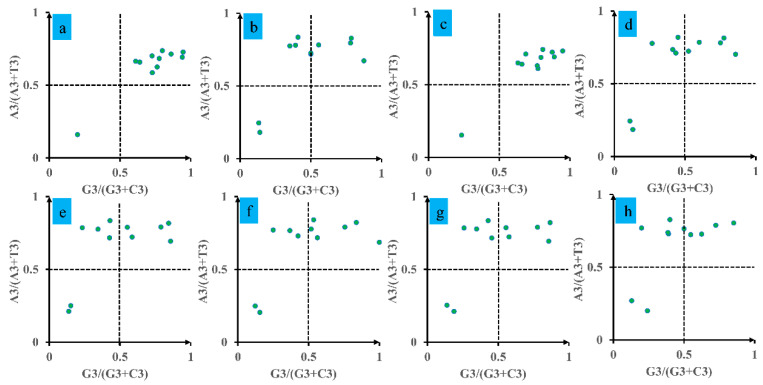
Parity Rule 2 (PR2) plot analysis of 11 core PCGs in 8 *Conidiobolus* strains. The 8 *Conidiobolus* species are *C. heterosporus*, *C. taihushanensis*, *C. rhysosporus*, *C. polytocus*, *C. lichenicola*, *C. polyspermus*, *C. mycophagus*, and *C. chlamydosporus* from (**a**–**h**).

An analysis using a Parity Rule 2 (PR2) plot was performed to evaluate biases in *Conidiobolus* mitochondrial genes (Figure 5). The plot, divided into four quadrants with axes centered on 0.5, showed a predominant clustering of data points for the eight *Conidiobolus* species in the first and second quadrants, indicating a pronounced preference for A over T. Notably, no data points were observed in the fourth quadrant, which signifies a bias towards T over A and G over C. These results suggest that factors such as natural selection play a significant role in shaping codon bias in *Conidiobolus* species.

### 3.7. Correspondence Analysis Results

**Figure 6:**
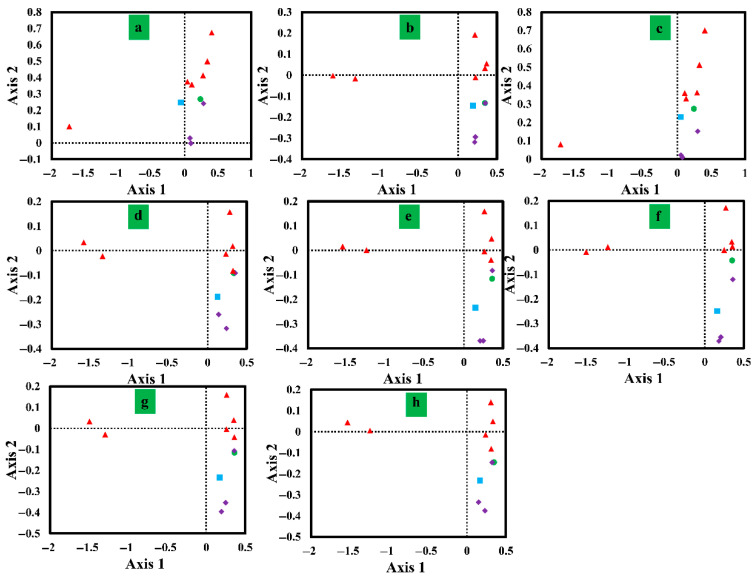
Correspondence analysis (COA) based on the relative synonymous codon usage (RSCU) values of 11 mitochondrial genes from 8 *Conidiobolus* strains. Purple represents the *cox* gene, red represents the *nad* gene, green represents the *atp6* gene, and blue represents the *cob* gene. The 8 *Conidiobolus* species are *C. heterosporus*, *C. taihushanensis*, *C. rhysosporus*, *C. polytocus*, *C. lichenicola*, *C. polyspermus*, *C. mycophagus*, and *C. chlamydosporus* from (**a**–**h**).

Correspondence analysis was performed on the Relative Synonymous Codon Usage (RSCU) values of mitochondrial genes in eight *Conidiobolus* species (Figure 6). Axis 1 was the primary source of variance, explaining 58.06% of the variance, followed by axis 2 (8.99%), axis 3 (7.38%), and axis 4 (3.89%). Axis 1 showed the highest variance contribution. The relationship between Axis 1 and GC, GC3s, ENC, CAI, CBI, and FOP was then evaluated. Significant correlations were found between axis 1 and CAI, CBI, FOP, GC, and GC3s across the eight *Conidiobolus* species, indicating a collective impact on the base bias of *Conidiobolus*. Additionally, notable distinctions were observed between the *nad2* and *nad6* genes and other core PCGs, suggesting differences in codon usage among core PCGs.

### 3.8. Optimal Codon Analysis

**Figure 7:**
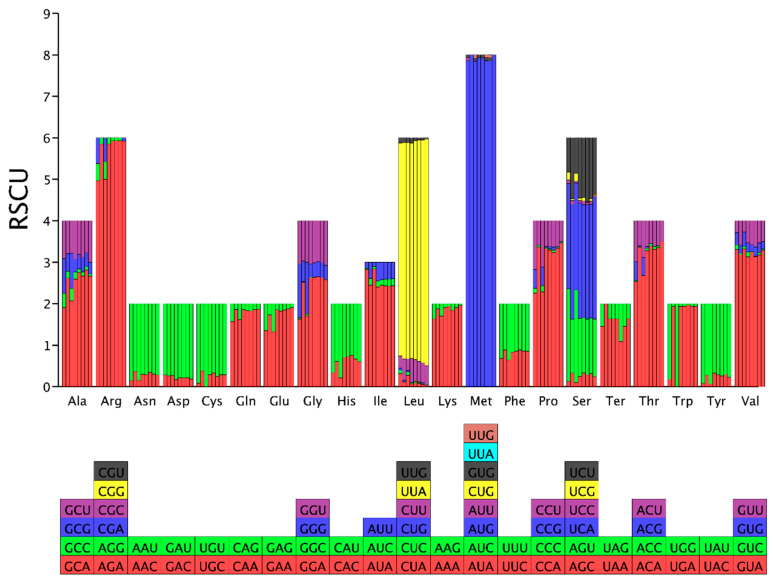
Relative synonymous codon usage (RSCU) analysis of 12 mitochondrial genes from 8 *Conidiobolus* strains. The color blocks with different colors on the bottom vertical axis represent different codons in the image above. The different letters in the horizontal axis represent abbreviations for different amino acids. The bar chart from left to right represents 8 *Conidiobolus* species, including *C. heterosporus*, *C. taihushanensis*, *C. rhysosporus*, *C. polytocus*, *C. lichenicola*, *C. polyspermus*, *C. mycophagus*, and *C. chlamydosporus*.

**Figure 8:**
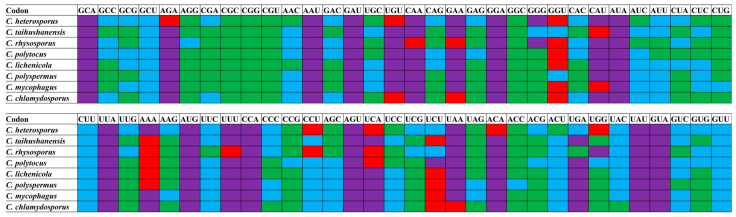
Optimal codons of 8 *Conidiobolus* strains (ΔRSCU > 0.08 and RSCU > 1), which are marked in red. Highly expressed codons (ΔRSCU > 0.08) were marked in blue and high-frequency codons (RSCU > 1) were marked in purple. Green represents general codons.

RSCU analysis revealed variations in the number of high-frequency codons (RSCU values > 1) among the eight *Conidiobolus* species, ranging from 18 to 25. Among these species, *C. heterosporus* had the fewest high-frequency codons, while *C. rhodosporus* had the highest. The codons GGG, CCU, and UGG were exclusively high-frequency in *C. heterosporus* and *C. rhodosporus*, suggesting differential codon usage in these species compared to others within the *Conidiobolus* genus. Examination of the 25 frequently used codons in *Conidiobolus* indicated that 9 ended with T, 13 with A, and only 3 with G, indicating a preference for A/T endings in *Conidiobolus* mitochondrial codons (Figure 7). Additionally, species-specific highly expressed codons (ΔRSCU > 0.08) were observed across the eight *Conidiobolus* species, highlighting distinct patterns in *C. heterosporus*, *C. taihushanensis*, *C. rhysosporus*, *C. polytocus*, *C. lichenicola*, *C. polyspermus*, *C. mycophagus*, and *C. chlamydosporus* (Figure 8). A comparative analysis identified optimal codons (ΔRSCU > 0.08 and RSCU > 1) in various species, predominantly A/T-ending codons. GGU was the most frequently utilized optimal codon in 6 *Conidiobolus* species, with AAA being the second most common in 5 species. Moreover, specific species exhibited unique optimal codons, including CAA, UUU, UAA, AGA, ACA, and UGG.

### 3.9. Phylogenetic Analysis Results

**Figure 9:**
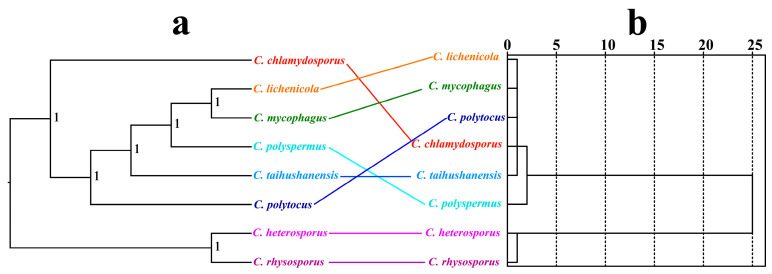
Relationship inference of different *Conidiobolus* strains based on the Bayesian inference (BI) (**a**) method and relative synonymous codon usage (RSCU) hierarchical clustering (**b**).

We compared the topology of the sequence-based phylogenetic tree (Figure 9a) with the RSCU similarity dendrogram (Figure 9b) to assess whether long-term evolutionary divergence correlates with contemporary patterns of codon usage, and to identify lineages where codon bias may have been shaped by distinct selective pressures. Bayesian inference (BI) was utilized to construct phylogenetic trees for eight *Conidiobolus* species based on a combined mitochondrial gene set. The analysis revealed two main evolutionary clades within the *Conidiobolus* species: one comprising *C. rhysosporus* and *C. heterosporus*, and the other including the remaining species (Figure 9a). Discrepancies in species relationships were observed when comparing phylogenetic relationships based on sequences with those based on Relative Synonymous Codon Usage (RSCU) (Figure 9b), particularly in the positioning of *C. polytocus*, *C. polyspermus*, and *C. chlamydosporus*. Nonetheless, the RSCU-based species relationships highlighted a distinct association among *C. rhysosporus*, *C. heterosporus*, *C. taihushanensis*, *C. mycophagus*, and *C. lichenicola*.

## 4. Discussion

The mitochondrial codon usage bias (CUB) in *Conidiobolus* species highlights natural selection as the primary evolutionary force shaping AT-biased synonymous codon preferences, which is consistent with the pattern of other fungal lineages [48,49,57]. Basidiomycota fungi like *Ganoderma* and *Amanita* exhibit moderate GC3 content (20–35%) and a stronger influence from mutational bias, as shown in recent comparative mitogenomic studies [48]. In contrast, *Conidiobolus* mitogenomes have high AT content (73.32%) and low GC3 content (13.40%), which represents the mitochondrial genetic characteristics of early-diverging fungi. This pronounced AT bias parallels observations in plant and animal mitochondrial genomes, where AT-rich environments favor translational efficiency of codons ending with A/T [58,59,60].

The weak correlation between GC12 and GC3 (slopes: 0.037–0.335) and the deviation of the effective number of codons (ENC) values from mutation-driven expectations in *Conidiobolus* stand in contrast to *Candida* mitogenomes, where mutation pressure predominantly shapes codon usage bias (CUB) [61]. This discrepancy underscores niche-specific selective pressures in Zoopagomycota, possibly associated with their diverse ecological roles—from obligate parasitism (*C. mycophagus*) to saprophytic decomposition (*C. polyspermus*). The phylogenomic incongruence between relative synonymous codon usage (RSCU)-based and sequence-based trees mirrors observations in *Ganoderma* and *Amanita* (Basidiomycota), where distinct selection pressures, rather than horizontal gene transfer (HGT), account for the discordant tree topologies [48].

PR2-bias analysis identified a consistent A > T and C > G bias in *Conidiobolus*. This bias is attributed to constraints on oxidative phosphorylation, promoting nucleotide asymmetry [62]. The presence of species-specific optimal codons, such as GGU (Gly) in six *Conidiobolus* species, mirrors findings in *Chlamydomonas reinhardtii* chloroplasts, where codon optimization improves translational accuracy during environmental stress [63]. Unlike *Candida* mitochondria, *Conidiobolus* does not exhibit GC3 heterogeneity, a feature attributed to tRNA modification systems that mitigate codon-anticodon mismatches [64], highlighting its distinct evolutionary path among fungi. *Bryophyte* mitogenomes with similar AT content display weak selection and mutation-driven Codon Usage Bias (CUB) [65], contrasting with *Conidiobolus* which shows strong selective pressures likely linked to ecological specialization. This parallels observations in *Laccaria bicolor*, where co-evolution between nuclear and mitochondrial genomes optimizes codon usage to enhance symbiotic efficiency [66]. However, *Conidiobolus*’ diverse ecological niche, including parasitism, saprophytism, and stress adaptation, may alleviate co-evolutionary constraints, leading to the promotion of lineage-specific codon usage strategies. The observed ENC_ratio_ (13.13–43.92%) and neutrality plot slopes are consistent with patterns observed in *Rhopalosiphum nymphaeae* (Aphididae), where selection predominates over mutation in highly expressed mitochondrial genes [67]. In *Conidiobolus*, the notable codon usage bias (CUB) in *nad4* (CAI: 0.052) and *nad6* (CAI: 0.226) may indicate differential expression or functional specialization, similar to findings in *Ophioglossum vulgatum* mitochondrial genes [68]. In summary, this paper analyzes the codon usage bias of early-diverging fungal mitochondrial genomes, providing a reference for understanding the genetic and evolutionary characteristics of the early-diverging fungal group.

## 5. Conclusions

While the weak GC12-GC3 correlation and the pronounced PR2 bias (A > T, C > G) are often interpreted as signatures of natural selection, caution is warranted in highly AT-rich systems like the *Conidiobolus* mitogenomes. The inherent amino acid composition of core mitochondrial proteins imposes strong constraints on GC12, potentially decoupling it from GC3 independently of selection on synonymous sites. Furthermore, strand-asymmetric mutation pressures common in mitochondrial DNA replication and transcription could generate the observed nucleotide biases (A/T and C/G asymmetry). Therefore, our results do not exclusively prove the dominance of selection but rather demonstrate that codon usage patterns are complex and likely shaped by an interplay of mutational biases, structural/functional constraints, and potential selective optimization for translational efficiency or accuracy. The phylogenomic incongruence and species-specific codon optimization observed highlight the intricate interplay of evolutionary pressures in ecologically diverse fungi. Future investigations should prioritize the examination of nuclear-mitochondrial interactions, variation in tRNA gene copy numbers, and adaptations in translational machinery to comprehensively elucidate the mechanisms of codon optimization in early-diverging fungi.

## Data Availability

The original contributions presented in this study are included in the article/Supplementary Material. Further inquiries can be directed to the corresponding authors.

## Supplementary Materials

The following supporting information can be downloaded at: https://www.mdpi.com/article/10.3390/jof12040231/s1, Figure S1: Variability of expected and actual ENC values of 11 mitochondrial genes from 8 *Conidiobulus* species.
