# Supplementary material for: Natural Selection Drives AT-Biased Codon Usage in Mitochondrial Genomes of Early-Diverging Conidiobolus Fungi (Zoopagomycota)
Source: J Fungi (Basel). 2026 Mar 24;12(4):231. doi: 10.3390/jof12040231 (PMC13118219; doi:10.3390/jof12040231)
Supplement: Supplementary file 1 [file jof-12-00231-s001.zip › jof-4093706-supplementary.pdf]

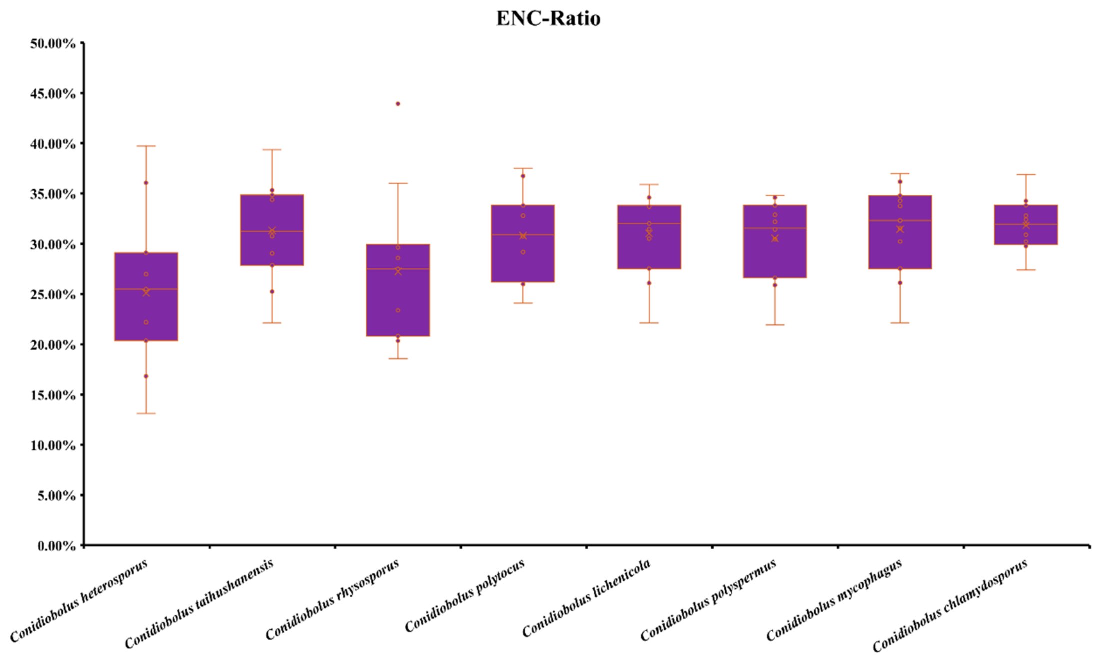

Figure S1 Variability of expected and actual ENC values of 11 mitochondrial genes from 8 *Conidiobulus* species.
